# Multiple Sclerosis patients carry an increased burden of exceedingly rare genetic variants in the inflammasome regulatory genes

**DOI:** 10.1038/s41598-019-45598-x

**Published:** 2019-06-24

**Authors:** Lovro Vidmar, Ales Maver, Jelena Drulović, Juraj Sepčić, Ivana Novaković, Smiljana Ristič, Saša Šega, Borut Peterlin

**Affiliations:** 1 Clinical Institute of Medical Genetics, Slajmerjeva 3, University Medical Centre Ljubljana, Ljubljana, Slovenia; 2 Clinic of Neurology, CCS, Faculty of Medicine, University of Belgrade, Belgrade, Serbia; 3 Postgraduate Study, School of Medicine, University of Rijeka, Rijeka, Croatia; 4 Faculty of Medicine, University of Belgrade, Institute of Human Genetics, 26 Visegradska, Belgrade, Serbia; 5 Department of Biology and Medical Genetics, School of Medicine, University of Rijeka, Rijeka, Croatia; 6 Division of Neurology, University Medical Centre Ljubljana, Zaloška 2, 1000 Ljubljana, Slovenia

**Keywords:** Genetics, Next-generation sequencing

## Abstract

The role of rare genetic variation and the innate immune system in the etiology of multiple sclerosis (MS) is being increasingly recognized. Recently, we described several rare variants in the NLRP1 gene, presumably conveying an increased risk for familial MS. In the present study we aimed to assess rare genetic variation in the inflammasome regulatory network. We performed whole exome sequencing of 319 probands, comprising patients with familial MS, sporadic MS and control subjects. 62 genes involved in the NLRP1/NLRP3 inflammasome regulation were screened for potentially pathogenic rare genetic variation. Aggregate mutational burden was analyzed, considering the variants’ predicted pathogenicity and frequency in the general population. We demonstrate an increased (p = 0.00004) variant burden among MS patients which was most pronounced for the exceedingly rare variants with high predicted pathogenicity. These variants were found in inflammasome genes (NLRP1/3, CASP1), genes mediating inflammasome inactivation via auto and mitophagy (RIPK2, MEFV), and genes involved in response to infection with DNA viruses (POLR3A, DHX58, IFIH1) and to type-1 interferons (TYK2, PTPRC). In conclusion, we present new evidence supporting the importance of rare genetic variation in the inflammasome signaling pathway and its regulation via autophagy and interferon-β to the etiology of MS.

## Introduction

Multiple sclerosis (MS) is a debilitating neurological disease affecting young adults. While its etiology remains unexplained, it is considered to be an autoimmune multifactorial disease with Epstein-Barr virus (EBV) infection as its main environmental risk factor^1^. Its genetic component has been addressed with considerable effort by the genome wide association (GWA) studies which have identified over two hundred risk conveying common genetic variants, predominantly related to the immune system genes^2^. However, GWA studies have proven inadequate for the investigation of exceedingly rare, and especially idiosyncratic variants which can only be detected using sequencing approaches^3–5^. We use the term “exceedingly rare” to refer to the variants with minor allele frequency (MAF) below 0.0001, representing a major part of human genetic variation which is not captured by the SNP-array genotyping technology^6^. Enabled by the next generation sequencing (NGS) technology, we have recently reported several rare variants in the NLRP1 gene, presumably conveying an increased risk for MS in families with multiple affected members^7^.

NLR Family Pyrin Domain Containing 1 (NLRP1) functions as the pattern recognition receptor (PRR) within the multi-protein signaling assemblies called inflammasomes, which lay at the forefront of the innate immune system. Their importance extends beyond infectious disease, as deregulated inflammasome activity has been associated with inflammatory^8^, autoimmune^9^ and neurological^10^ diseases, including MS^11–14^. Various types of inflammasomes are distinguished based on the involved PRR. We focus on NLRP1/NLRP3 inflammasomes which operate in a similar fashion. Canonical inflammasome activation requires a priming step which promotes the transcription and translation of core inflammasome proteins: NLRPs and caspase-1 (CASP1)^15^. Thereafter, a wide variety of pathogen or danger associated molecular patterns (PAMP/DAMP) recognized by the NLRPs can trigger their oligomerization with Apoptosis-Associated Speck-Like Protein (PYCARD) to assemble an active inflammasome complex able to process its main effectors, interleukin-1β (IL-1β) and IL-18, whose increased levels are associated with MS^16,17^, demyelination and blood-brain barrier breakdown^18^. The priming step can be triggered by a number of independent PRRs and is largely propagated by Nuclear Factor Kappa-B (NF-kB) and type-1-interferon (IFN1) responses^19^. To curb excessive inflammasome activation the cell employs several negative regulatory mechanisms, many of which have evolved recently and are unique to humans. These include the sequestration of inflammasome proteins^20^ and their posttranslational modifications leading to their elimination by autophagy^21,22^. Examples of crosstalk between these regulatory modalities were described recently^23,24^, adding additional complexity to the network of proteins governing inflammasome activation.

While the sequencing of extended families with multiple affected members has been successful at identification of rare causal variants in the past, this approach is generally limited to highly penetrant variants which segregate with the disease. On the other hand, association studies are required to identify novel variants with small to moderate effects on disease risk. To address the diminishing power that comes with increased rarity of the investigated variants, variants can be aggregated^25^ over a biologically relevant region, such as a gene or a pathway. The burden of these variants can be evaluated for an association with the disease as a set^25^, instead of testing the effects of individual variants, as is commonly done in GWA studies.

Based on existing evidence^11–14^ and our previous results^7^ we hypothesized that rare functional variants at inflammasome related loci can deregulate inflammasome function, leading to increased risk for MS. In the present study we aggregated the NGS-discovered rare protein-altering variants over 62 genes crucial for NLRP1/NLRP3 inflammasome regulation (Table 1) and compared their overall burden among the cohorts of MS patients from multiplex families (MSFAM), sporadic MS patients (MSS), and controls (CTRL). The variants’ MAF was obtained from the gnomAD^26^ database of over 140,000 sequenced individuals, and their potential for functional consequence on the proteins was assessed by CADD^27^ scoring algorithm.

**Table 1:** The inflammasome regulation gene panel. Priming step genes include cytosolic DNA/RNA sensors and their key downstream signaling mediators^19^. Protein tyrosine phosphatases (PTP) act through phosphorylation of NLRP3^85^ or PYCARD^86^ but are written in a separate column for the sake of clarity. Five PTP genes already associated with inflammatory disease^87^ were included in the panel. Common protein names are written in the parenthesis.

Essential inflammasome	Inflammasome priming^19^		Regulation by sequestration^28^	Postranslational modification and auto/mitophagy^28^		PTPs^28^
**NLRP1**	EIF2AK2 (PKR)	IRF3	HDAC6	BRCC3	NOD2	PTPN2
**NLRP3**	DDX58 (RIG-1)	IRF7	NR1H4	MARCH7	RIPK2	PTPN6
**PYCARD**	IFIH1 (MDA-5)	TYK2	CASP8	FBXL2	SQSTM1 (P62)	PTPN22
**CASP1**	DHX58 (LGP2)	JAK1	FADD	MEFV	SESN2	PTPRC
	TMEM173 (STING)	MAVS	POP1	ULK1	IL4	DUSP1
	ZBP1 (DAI)	STAT1	PYDC2 (POP2)	TRIM31	SIRT2	
	POLR3A	STAT2	PYDC5 (POP3)	MAPK8	FLI1	
	CGAS	TICAM1	CARD16 (PSEUDO-ICE, COP-1)	ATG5	LRRFIP2	
	LRRFIP1	MYD88	CARD17 (INCA)	BECN1		
	TBK1	CHUK (IKBKA)	CARD18 (ICEBERG)	ATG16L1		
	IKBKE		HSP90AA1^88^	MAP1LC3B		
			HSP90AB1^88^	FOXO3		

The results presented in this study demonstrate an increased burden of rare protein altering variants in the inflammasome regulatory genes of MS probands. The excess burden is especially pronounced for variants ranking highest in the employed pathogenicity criteria.

## Results

In the investigated inflammasome regulatory genes^28^ (Table 1), we detected 300 rare (MAF < 0.05) protein altering variants with total allele count of 1031. The variant loci were successfully genotyped in 99.7% of probands.

SKAT burden test^29^ showed both familial and sporadic MS cohorts were significantly enriched for rare variant burden in the analyzed gene panel compared to the control cohort (MSFAM: p = 0.00025; MSS: p = 0.00002; MS-combined: p = 0.00004).

**Figure 1:**
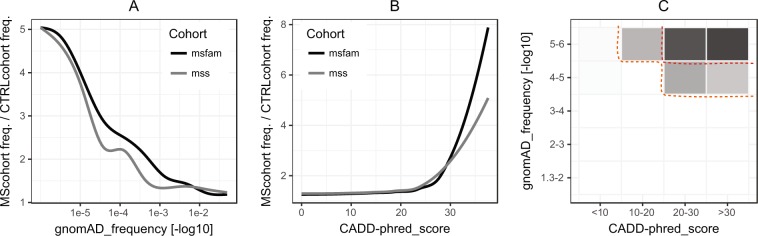
Variant burden by MAF and CADD scores. Plots (**A** and **B**) represent how the burden of variants in the MS cohorts (relative to the control cohort; normalized to cohort sizes) changes with different gnomAD MAF and CADD score cutoffs, respectively. Panel C jointly accounts for both variant attributes and displays the relative burden of both MS cohorts combined. Variants qualifying into the top right corner (inner dotted line) were found almost exclusively among the MS probands and are listed in the Table 2 (*selected* variants). All variants qualifying within the outer dotted line were 4.7 times more common among the MS probands and are available in the Supplementary Table S1.

In a separate analysis performed to identify a subset of variants with the most likely functional significance, all variants were annotated with CADD scores and MAF obtained from the gnomAD database. Variants which were absent from the gnomAD database were five times as common among the MS probands compared to the ethnically matched control cohort (Chi.sq p = 0.008). Plotting the aggregated variant burden for multiple MAF and CADD-score cutoffs demonstrated a trend whereby increasingly rare (Fig. 1 - panel A) and damaging (Fig. 1 - panel B) variants were more enriched in both MS cohorts (individually compared to the control cohort). To assess if this finding was specific for the investigated set of inflammasome regulatory genes (Table 1), the observed trends were compared to 100,000 random gene sets of the same size in a Monte Carlo simulation. The results demonstrated that the trends observed for the selected inflammasome gene panel were significant and could not be explained by a cohort related dataset bias (MAF analysis: p = 0.0005; CADD analysis: p = 0.017; see Supplementary Fig.S1 for results of individual MS cohorts).

In addition to separately addressing the MAF and CADD criteria, an even more convincing enrichment pattern was unveiled when both variant rarity and predicted pathogenicity were accounted for concurrently (Fig. 1; panel C). Missense variants with MAF below 1 × 10^−5^ (corresponds to none, one, or two variant instances in the gnomAD database) and CADD-score above 20 represented 10% of missense variants. These variants were present almost exclusively among the MS probands and are hence forth referred to as the “*selected*” variants (Table 2). Some variant types (stop-gain/loss, splice-site, and frame-shift) are known to commonly disrupt protein functionality. Interestingly, among the 20 such variants discovered, the rarest 10 were found exclusively in the MS probands. These variants were included among the *selected* variants using the same criteria, except for the frame-shift variants which could not be assessed by the CADD algorithm and qualified solely based on the MAF criterion (three variants).

**Table 2:** *Selected* variants. “hg19” represents genome coordinates in a “chr_coordinate_reference-allele_alternate-allele” form. Splice site variants have transcript information reported in the “Protein_change” column. “gAF” and “gAC” columns represent variant frequency and allele count reported in the GnomAD database and “MS” and “CTRL” columns report the absolute numbers of variant alleles in the combined MS and control cohorts, respectively. All *selected* variants were present only in the heterozygous state, both in our cohorts and gnomAD (if present). Variants marked with * in the gene column also fulfilled the MS enriched variants criteria. “Freq.” is the overall frequency of the *selected* variants’ alleles considering the MS-combined and control cohort sizes of n = 175 and n = 144, respectively. Other relevant annotations are available in the Supplementary Table S1.

GENE	hg19	P.change	Var.type	gAF	gAC	CADD	MS	CTRL
***Selected*** **variants**								
CASP1	11_104901069_A_-	p.Asn205fs	frameshift	4.10E-06	1	NA	1	0
CASP8	2_202131331_C_A	p.Ala100Asp	missense	NA	0	24.4	1	0
DHX58	17_40257124_G_C	p.Thr438Arg	missense	4.07E-06	1	26.7	1	0
EIF2AK2	2_37336412_T_A	p.Glu468Asp	missense	NA	0	25.4	1	0
FOXO3	6_108985199_A_G	p.Asp388Gly	missense	4.06E-06	1	23.3	1	0
HDAC6	X_48681075_A_G	p.Thr795Ala	missense	NA	0	25.5	0	1
HSP90AB1	6_44220815_T_G	p.Cys589Gly	missense	4.08E-06	1	22.3	1	0
IFIH1*	2_163134114_T_C	p.Met619Val	missense	8.14E-06	2	24.4	2	0
MEFV	16_3304478_-_C	p.Gln198fs	frameshift	NA	0	NA	1	0
NLRP1	17_5445285_T_-	p.Asn864fs	frameshift	NA	0	NA	1	0
NLRP3	1_247588351_C_A	p.Leu536Met	missense	NA	0	24	1	0
POLR3A	10_79741230_C_T	p.Asp1283Asn	missense	8.13E-06	2	35	1	0
POLR3A	10_79769710_C_T	p.Arg561Gln	missense	8.12E-06	2	35	1	0
POLR3A	10_79745740_G_A	p.Arg998Cys	missense	8.12E-06	2	25.1	1	0
POLR3A	10_79753041_T_G	p.Ile901Leu	missense	NA	0	22.6	1	0
POLR3A	10_79770247_T_G	p.Ile542Leu	missense	NA	0	25.2	1	0
POLR3A	10_79764601_T_C	p.Gln707Arg	missense	4.06E-06	1	22.6	1	0
POP1	8_99161077_C_T	p.Pro582Leu	missense	NA	0	33	1	0
PTPN2	18_12794395_C_T	p.Arg377Gln	missense	NA	0	27.2	1	0
PTPN22	1_114399219_G_A	p.Ala144Val	missense	NA	0	22.1	1	0
PTPRC	1_198701436_C_T	p.Pro661Leu	missense	4.08E-06	1	34	1	0
PTPRC	1_198665899_C_A	p.His53Gln	missense	NA	0	22.9	1	0
RIPK2*	8_90782096_C_T	p.Pro194Ser	missense	NA	0	32	2	0
SIRT2	19_39371539_T_C	c.748-2 A > G	splice_accep	NA	0	20.6	1	0
SIRT2	19_39380575_G_A	p.Pro99Ser	missense	4.06E-06	1	25.7	1	0
TYK2	19_10468526_C_T	p.Ala794Thr	missense	NA	0	23.1	1	1
TYK2	19_10479064_G_A	p.Ala75Val	missense	8.12E-06	2	25.3	1	0
TYK2	19_10468793_C_T	p.Gly733Ser	missense	4.21E-06	1	25	1	0
ULK1	12_132396530_C_T	p.Pro331Leu	missense	8.15E-06	2	25.7	0	1
						**Freq**.:	0.08	0.01

**Table 3:** MS enriched variants. See Table 2 caption text for header description. Columns “gAC”, “MS”, and “CTRL” report the total allele count while the allele count contributed by the homozygous probands is reported in the brackets. Variants Pro194Ser and Met619Val in RIPK2 and IFIH1 genes fulfilled both MS enriched and *selected* variants criteria and are reported in the Table 2.

GENE	hg19	P.change	Var.type	gAF	gAC	CADD	MS	CTRL
CARD18	11_105009760_C_A	p.Gly18Val	missense	1.63E-05	4	11.83	3	0
CHUK	10_101959733_G_A	p.Pro575Leu	missense	1.63E-05	4	24.4	2	0
DHX58	17_40257832_CTG_-	p.Gln391del	inframe_del	4.11E-06	1	NA	2	0
DUSP1	5_172197790_C_T	p.Ala56Thr	missense	0.022276	2192(36)	22.7	12	6
HDAC6	X_48681187_G_A	p.Arg832His	missense	0.023789	4046(39)	23.9	17(14)	7(4)
IFIH1	2_163134090_C_A	p.Glu627*	stop_gained	0.003199	786(1)	38	7	1
IL4	5_132015548_G_A	p.Arg109Gln	missense	7.76E-05	19	14.77	2	0
LRRFIP1	2_238671578_T_C	p.Ser408Pro	missense	2.44E-05	6	2.929	2	0
CGAS	6_74161604_C_T	p.Gly101Arg	missense	0.015178	2080(25)	8.006	11	5
NLRP1	17_5424908_C_T	p.Arg1240His	missense	4.47E-05	11	0.108	3	0
PTPRC	1_198718604_G_A	p.Asp1000Asn	missense	0.000193	47	28.5	3	1
SIRT2	19_39379770_C_T	p.Arg153His	missense	0.008683	2129(19)	34	9(2)	3
TBK1	12_64891037_G_C	p.Glu653Gln	missense	0.000254	56	18.96	2	0
TICAM1	19_4816670_G_C	p.Leu574Val	missense	0.000138	34	0.001	2	0
ZBP1	20_56195349_C_T	p.Met1?	start_lost	0.014182	3042(32)	13.4	11	4

We detected 17 variants present in multiple MS probands which were considered *MS enriched* (Table 3), given the over-representation of their alleles in the MS cohorts compared to their MAF reported in gnomAD database (Fisher exact test; p < 0.01). Using the same method, two variants whose alleles were also overrepresented in our control cohort were considered population specific and were not included among the *MS enriched* (or *selected*) set of variants. *MS enriched* variants with CADD-phred scores above 20 were discovered in genes RIPK2, IFIH1, CHUK, PTPRC, HDAC6, DUSP1, and SIRT2.

The current study replicated two variants in the NLRP1 gene (Thr670Ile and Phe274Leu) which were recently described in MS probands by Bernales *et al*.^30^ - a study aimed to replicate the results of our previous publication^7^. Considering the combined sample sizes and the rarity of the variant alleles in the gnomAD database, the replication was significant as by our enrichment analysis described above (also see methods – Statistical analysis section).

All variants discovered in this study and their variant call quality scores are available with additional annotations in the Supplementary Table S1.

## Discussion

**Figure 2:**
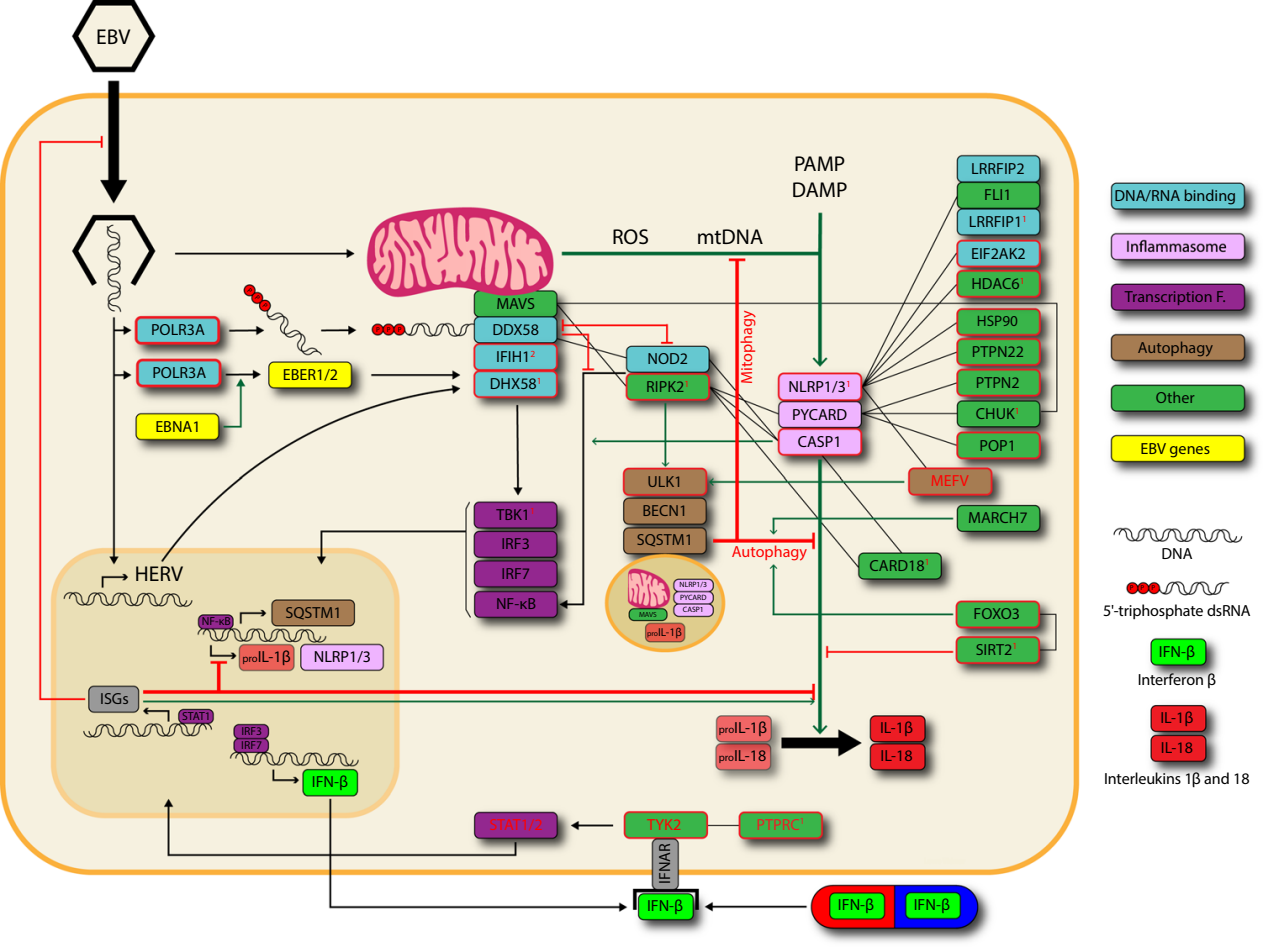
Inflammasome regulatory network. The depicted genes carried variants with MAF and CADD scores falling within the outer dotted region in Fig. 1; panel-C. Genes carrying the *selected* variants (inner dotted region in Fig. 1; panel-C) are bolded in red. Numbers next to the gene names represent the number of MS enriched variants. Black, green and red arrows represent general pathways and stimulatory/inhibitory effects, respectively. Genes stacked together or connected with black lines form complexes or have confirmed protein-protein interactions (in the majority of cases via the CARD protein domain)^53^. Genes written with red have already been associated with MS. The genes are organized in five groups: Interferon response, RNA polymerase 3 and rig-like receptors (RLRs), main inflammasome genes, other genes (regulating inflammasome activity by sequestration or phosphorylation), and the NOD2/RIPK2 complex integrating the latter three and inducing the autophagy pathway. EBV – Epstein-Barr virus, PAMP/DAMP – Pathogen/Damage-associated molecular patterns, ROS – Reactive oxygen species, HERV – Human endogenous retroviruses, ISGs – Interferon-stimulated genes, IFNAR – Interferon receptor.

In the present study we have discovered a significantly increased burden of rare protein-altering variants within the genes involved in inflammasome regulation among the patients with MS. The increased burden was most pronounced for the exceedingly rare variants with concomitantly high predicted pathogenicity estimates which were present in several genes and pathways with already established links to MS or its risk factors (Fig. 2).

While GWA studies have been very successful in the identification of common (MAF > 0.05) MS risk conveying variants, their design has proved inadequate for capturing the contribution of rare variants, mainly for two reasons. Firstly, the employed SNP-array genotyping technology requires each variant to be anticipated and is therefore unable to detect previously unknown variation. And secondly, the power of association studies focusing on individual variants declines with variant rarity^25^. An attempt to address the former has been published very recently and employed the new Illumina exome arrays containing probes for rare protein altering variants^6^. Nevertheless, variants below MAF 0.0001 received negligible coverage and even with nearly 70,000 involved cases and controls their main results were limited to 7 moderately rare (MAF 0.002 to 0.056) newly associated MS variants in six different genes. Interestingly however, two of them and one paralogue (TYK, EIF2AK2, and HDAC6) also contained *selected* variants in our study and further two (NLRP8, PRF1) are known to be important for the inflammasome function^31^, corroborating the results of our present study. Similarly, the role of rare variation in the inflammasome pathway and TYK gene was supported by previous MS studies employing the NGS technology^32,33^.

In the present study, we employed variant aggregation^25^ and MAF variant annotations obtained from GnomAD, which enabled us to study the exceedingly rare variation despite our smaller sample size. The significant enrichment of newly discovered variants (absent in gnomAD) among the MS cohorts compared to the ethnically matched control cohort was supported by significant result of the SKAT burden test and the Monte Carlo simulation analysis. Furthermore, among the exceedingly rare variants, those also predicted to be damaging to the protein function showed the most pronounced enrichment (Fig. 1 – panel C).

Besides in the aggregated variant burden analysis, we also used gnomAD to assess the significance of several rare variants which were discovered in two or more MS probands (*MS enriched* variants). While we acknowledge that these variants do not achieve genome-wide significance levels set for associating individual variants, the presence of these variants in multiple MS probands does represent an additional independent criterion. We thus consider the *selected* variants which were also *MS enriched* (RIPK2 p.Pro194Ser and IFIH1 p.Met619Val) our best candidates to be confirmed as MS associated in the future studies. However, as additional affected and healthy members from the extended MS families were not systematically available for segregation analysis, the penetrance of these variants was not assessed in the present study.

Our results provide novel evidence for the importance of rare variation in the inflammasome pathway for the MS etiology. Similarly, the importance of exceedingly rare variation was recently demonstrated for other CNS diseases, such as epilepsy^34^ and schizophrenia^35^. In general, the advent of NGS revealed that exceedingly rare variation accounts for a major part of human genetic variation^26^, thus strengthening the rationale for new studies and approaches aimed at the investigation of their effects on multifactorial disease, including MS.

Our approach has identified a set of of *selected* exceedingly rare variants with high pathogenicity predictions (CADD score), which appeared in several functionally highly connected genes, suggesting the importance of molecular mechanisms linking EBV, interferon-β signaling, and autophagy to the etiology of MS (Fig. 2). The highest burden of *selected* variants was discovered in the active subunit of RNA polymerase III (POLR3A), despite the gene being underrepresented with missense variation in the general population^26^. The activity of POLR3 has been demonstrated as crucial for the immune response against the EBV and other DNA viruses^36^. Its function is mediated by the RIG-1-like receptors (RLRs)^36,37^ which bind to Mitochondrial Antiviral Signaling protein (MAVS) to elicit an IFN1 response. RLRs also contained several variants which arguably affect the protein function - the exceedingly rare start-lost and stop-gain variants (p.Met1? and p.Arg575*) in the DDX58 (a.k.a. RIG1), a missense variant (p.Thr438Arg) in the DHX58 and a missense (Met619Val) and stop-gain (p.Glu627*) variants present in the IFIH1 (a.k.a. MDA-5) gene of 2 and 7 MS probands, respectively. Some of these have been reported previously - The IFIH1 variant p.Glu627* has been associated with lower poly(I:C)-induced IFN1 production^38^ and the variant POLR3A p.Gln707Arg with severe varicella zoster virus (VZV) infections^39^. We therefore hypothesize that the variants described in the POLR3-RLR pathway could alter the response to viral infections in MS patients, particularly VZV and EBV which are known risk factors for MS^40^. Besides their importance in exogenous infections, RLRs also respond to human endogenous retrovirus (HERV)^41^ sequences whose increased expression is associated with MS^42^ and can also be induced by the infection with EBV^43^. The IFN1s resulting from the activation of RLRs act in an auto- and paracrine fashion to promote clearance of infection, inflammasome priming and its inactivation^44,45^. Crucial to IFN1 pathway are the TYK2 and PTPRC^46^ genes which have been associated with MS in the GWA studies^47^ and contained several *selected* variants in the present study. IFN1 (intereferon-β) represents the mainstay of MS therapy and its beneficial effects have already been attributed to its inhibitory effect on inflammasome activity^13,14,45,48^. While it is reasonable to assume that MS patients with defective POLR3-RLR driven production of interferon-β would respond well to external supplementation, it is tempting to speculate that those with affected TYK2 pathway might represent the non-responders.

The highest CADD-scored absent-in-gnomAD variant in the present study was discovered in the RIPK2 gene (p.Pro194Ser). The variant was discovered in two MS probands and its extremely high pathogenicity predictions are corroborated by the importance of proline residues for RIPK2 protein folding^49^. Moreover, RIPK2 has reduced tolerance to both missense and loss of function variants based on ExAC functional constraint data^26^. RIPK2 is essential for the function of NOD2, with whom it forms a protein complex. While commonly associated with response to bacterial infection, NOD2 is increasingly recognized in response to both RNA and DNA viruses^50–52^. By interacting with both the DDX58 and MAVS on the one hand^53^ and with CARD18, PYCARD and CASP1 on the other^54^, the NOD2/RIPK2 complex is centrally positioned to serve as the integrator of both pathways. This is supported by the recent discovery of mutually antagonistic action between the DDX58 and NOD2^55^. The suggested mechanism is based upon the sequestration of DDX58 and NOD2 away from MAVS and RIPK2, which are necessary for downstream IFN1 and NF-kB signaling, respectively^55^ (Fig. 2). RIPK2’s ability to engage in NF-kB signaling is also affected by its interaction with CASP1, and hypomorphic genetic variants in the latter have been suggested as the cause of febrile episodes^56^. Interestingly, we discovered an exceedingly rare missense variant (CASP1 p.Arg45Ser) at amino acid residue deemed important for this interaction^57^.

RIPK2 has been shown to dampen inflammasome activation via the regulation of NF-kB-ULK1-SQSTM1 mediated mitophagy during viral infection^58,59^. Both autophagy and mitophagy are important negative regulators of inflammasome activation^22^. Interestingly, RIPK2, along with the other members of the RIP kinases family, is emerging as an important regulator of the innate and adaptive immune system^60^. Furthermore, its importance in the context of MS has already been demonstrated in both human and animal studies^61–63^, and clinical studies on MS patients involving RIPK2 inhibitors^64^ have already been suggested^65^. Several other genes involved in autophagy contained the variants *selected* in our study. Both SIRT2 and FOXO3 were involved in inflammasome inactivation via autophagy in murine model of nonalcoholic fatty liver^66,67^, which could be relevant in the context of MS as childhood obesity is a recognized MS risk factor^68^. One possible mechanism could be the reduced activity of SIRT2 in high fat diet, resulting in increased trafficking of NLRP3 to the mitochondria and subsequent inflammasome activation^69^. Additionally, SIRT2 also plays a critical role in oligodendrocyte differentiation and the expression of myelin-specific genes^70^, and deacetylates FOXO3 in response to oxidative stress and caloric restriction^71^. Importantly, MEFV has recently been shown to recruit and organize key components of autophagic machinery, including ULK1 and BECN1, and to act as autophagic receptor specific for NLRP1, NLRP3 and CASP1^72^.

While all the *selected* variants were in heterozygous state, several genes carrying the reported variants have reduced tolerance to loss of function (RIPK2, CASP1, NLRP3, PTPRC) and missense variation (POLR3A, RIPK2, HDAC6, NLRP3, TYK2). Additionally, due to the nature of cooperative inflammasome assembly^73^, involving the interactions of many parts and regulatory proteins, the presence of dominant negative mutations and gene dosage sensitivity is likely^74,75^. As the regulation of both RLRs and inflammasome proteins rely on protein-protein interactions via the CARD protein domain, the final output of the signaling complex could be altered by rare functional variants affecting these interactions. Such reasoning can potentially explain increased production of IL-1β caused by presumably hypomorphic variants in the NLRP1 gene (p.Gly578Ser and p.Asn864fs), which we observed in the previously performed functional studies^7^. All genes from our inflammasome panel which have previously been associated with MS in GWA studies^2,47^ mediate the interferon-β pathway and contained exceedingly rare variants in the present study (TYK2, STAT1, and PTPRC). These genes provide possible examples of MS heritability being conveyed by common as well as rare variation within the same genes.

On Fig. 2 we provide a review of inflammasome regulatory pathway and highlight the contribution of two pathways in which we found the *selected* variants. Firstly, while the afferent arm of interferon-β signaling pathway has already been associated with MS in GWA studies, we provide preliminary evidence for the importance of its efferent arm which leads to interferon-β production in response to infection with EBV and increased expression of HERV. As the EBV infection was recently demonstrated in the brain of 90% of MS patients where it extended beyond its canonical host (B-cells) to include astrocytes and microglia as well^76^, the POLR3-RLR pathway could also play a role in cells without clear autoimmune function, including the maintenance of the blood-brain barrier. Secondly, our research corroborates the importance of auto/mito-phagy for inflammasome regulation as we highlight several pivotal genes involved in the process – RIPK2, MEFV and SIRT2, FOXO3. The former two have already been associated with MS in animal studies and family based rare-variant association studies, respectively, and the latter two were implicated in the inflammasome inhibition following high fat diet. As childhood obesity, and EBV infection are known risk factors for MS, it is possible that these environmental effects might act in synergy with genetic predisposition, including in the form of rare coding variants with functional effects described within the inflammasome pathway in the present study.

In conclusion, we discovered significantly increased rare variant burden in the inflammasome regulating genes of patients with MS, which was most prominent for the exceedingly rare variants that were scored highly by the pathogenicity prediction algorithms. These variants support the overall importance of inflammasome and its regulation by interferon-β and auto/mito-phagy to the etiology of MS.

## Methods

### Ethical statement

The study was performed in accordance with the principles stated in the Declaration of Helsinki. All participants gave informed written consent to participate in the study. To maintain confidentiality, their names were replaced with proband IDs at blood draw. The study was approved by the National Medical Ethics Committee (#90/08/12).

### Participants

The study involved 319 subjects categorized into 3 cohorts. The MSFAM cohort included 86 MS probands from independent families with multiple affected members (2 or more 1^st^ degree relatives with MS). Only a single patient with MS per family was included in the study. The sporadic (MSS) cohort included 89 MS patients without family history of the disease. The diagnosis of MS was established in accordance with the McDonald criteria^77^. The MS probands originate from Slovenian, Croatian and Serbian population. Ethnically matched patients referred to our institute for the diagnosis of suspected Mendelian disease unrelated to MS were used as controls (CTRL cohort, n = 144). The cohorts had an average age of 43 and sex ratio of 1.6:1, female to male, both of which did not significantly differ between the cohorts. Nineteen probands previously screened solely for the NLRP1 variants in our previous publication^7^ were included in the present MSFAM cohort.

### Whole exome sequencing (WES), raw data analysis, and variant annotation

WES of whole blood derived DNA was performed on Illumina HiSeq-2000 platform to a standard depth of coverage of 30x. Read sequences were aligned to hg19 reference genome using Burrows-Wheeler (BWA) aligner and processed in accordance with genome analysis toolkit^78^ (GATK) best practices (haplotyper-caller in GVCF mode with subsequent joint genotyping and variant filtering with VQSR)^79^. Variant call quality scores assigned by the VQSR are reported in the Supplementary Table S1. Various exome capture kits were employed for NGS library preparations (Agilent-All-Exon 2/5/6 and Illumina Nextera-Exome) with matching proportions between the cohorts. To further diminish the possibility of capture kit derived bias, only universally captured loci were analyzed, and only variants that were successfully genotyped in over 95% of probands were retained. A BED file representing the overlap of capture kits was generated using Bedtools^80^ to limit the GATK analysis downstream of alignment step to universally captured loci. Additionally, all *selected* and MS enriched variants had BAM alignment files manually inspected in the Integrative Genomics Viewer (IGV)^81^ to further reduce the possibility of false positive variant calls. Variants were annotated with snpEff and ANNOVAR within the Variant Tools^82^ software package. Genome Aggregation Database (gnomAD) was employed as the source of variant frequencies in worldwide populations (http://gnomad.broadinstitute.org/)^26^. Combined Annotation–Dependent Depletion (CADD)^27^ pathogenicity prediction algorithm was used to estimate variants’ effect on protein function. CADD-phred is a normalized logarithmic scale according to which the variants with scores above 10, 20 and 30 represent the top 10, 1, and 0.1 percent of highest ranking variants^27^. Synonymous variants, variants outside coding regions, and variants with MAF > 0.05 in gnomAD or within the entire dataset were filtered out, leaving only rare protein-altering variants for all further statistical analysis.

### Gene panel selection

The panel was assembled out of genes coding for essential inflammasome constituents (NLRP1/3, PYCARD and CASP1) and genes involved in the regulation of its activation^28^. The latter were classified into: a) Genes involved in the priming step leading to transcription of inflammasome constituents, b) inhibition of inflammasome assembly by their sequestration, and c) post-translational modification and their removal via autophagy or mitophagy. The final gene panel included 62 genes listed in the Table 1.

### Statistical analysis

Statistical analysis was performed in R statistical environment^83^. Variants within genes from the analyzed panel were aggregated and their burden compared pairwise between MS and control cohorts. The significance of the difference in the observed rare variant burden was assessed using R package “SKAT“^29^. “SKATBinary” test in “burden” mode was performed with variant weighting based on gnomAD variant frequency data, as described by Madsen et Browning^84^. For the purpose of variant weighting, the variants absent in gnomAD database were assigned a gnomAD MAF of 1 × 10^−6^ as by extrapolation (variants with 2 and 1 alleles reported in gnomAD have MAFs of 8 × 10^−6^ and 4 × 10^−6^, respectively). As the SKAT test was performed three times (control cohort against both of the MS cohorts and a combined MS cohort) we set a Bonferroni corrected (Alpha: 0.01) p-value of 0.003 as the cutoff for statistical significance. Pearson’s Chi-squared test with Yates’ continuity correction was performed to determine if the variants absent in gnomAD were significantly overrepresented among the MS probands compared to the control cohort (using the total number of variants identified in each cohort as the background). To establish the MAF and CADD criteria for the *selected* most likely causative missense variants, the inter-cohort variant burden comparison was performed at 300 equally distributed MAF and CADD-score cutoffs below and above which the variants were considered for analysis, respectively (Fig. 1; panels A and B). The cutoff of CADD = 20 was chosen as above this value the overrepresentation of variants among the MS probands started to grow exponentially. Together with a cutoff at MAF <1 × 10^−5^ these criteria selected 10% of all identified missense variants. All variants with CADD scores available (including stop-gain/loss and splice-site variants) qualified among the selected variants using the same criteria. Frame-shift variants could not be assessed by the CADD algorithm and were included based only on their MAF. To assess the significance and the specificity of the trends depicted in Fig. 1 (panels A and B) to our chosen inflammasome gene panel, linear regression analysis was performed, and the slope coefficients were compared to coefficients obtained for 100,000 random gene panels of the same size. More information on the method is available in the Supplementary File).

Variants which were discovered in 2 or more MS probands were evaluated to determine whether their multiple occurrence in our MS cohorts might be significant, considering their relative scarcity in the general population (gnomAD). Fisher exact test was performed comparing the proportions of variant/reference alleles in our MS cohorts and the gnomAD database. To eliminate populationally specific variants, the same test was performed comparing our control cohort to gnomAD. Variants attaining nominal significance (p < 0.01) in the former but not in the latter test were considered “*MS enriched”*. The same procedure was performed to estimate the significance of the replication of the two variants reported by Bernales *et al*.^30^. As both variants were reported in the same proband (in both studies) and have nearly identical frequency reported in the gnomAD, they were considered as a single haplotype and a single Fisher test comparing the proportions of variant/reference haplotypes in MS cohorts from both studies and the gnomAD database was performed.

## Supplementary information

Supplementary information

Supplementary Table S1

## Data Availability

All data analyzed during this study are included in this published article and its Supplementary Information files. All variant datasets generated during the current study are available from the corresponding author on reasonable request.
