# Supplementary material for: Multiple Sclerosis patients carry an increased burden of exceedingly rare genetic variants in the inflammasome regulatory genes
Source: Sci Rep. 2019 Jun 24;9:9171. doi: 10.1038/s41598-019-45598-x (PMC6591387; doi:10.1038/s41598-019-45598-x)
Supplement: Supplementary file 1 — Supplementary information [file 41598_2019_45598_MOESM1_ESM.docx]

**Multiple Sclerosis patients carry an increased burden of ultra-rare genetic variants in the inflammasome regulatory genes**

## Lovro Vidmar, Aleš Maver, Jelena Drulović, Juraj Sepčić, Ivana Novaković, Smiljana Ristić, Saša Šega, Borut Peterlin

## Supplementary information

For the variants discovered within the selected inflammasome gene panel, our analysis demonstrated a trend observed when gnomAD minor allele frequencies (MAF) and CADD scores were used as predictors of variant allele overrepresentation within the MS cohorts compared to the ethnically matched control cohort (Figure S1 - panels A and B). The analysis accounted for the difference in cohort sizes.

The following analysis was performed to assess if these trends were specific to the selected inflammasome gene panel. Linear model was fitted using the function "lm()" from the R "stats" package to reveal the slope coefficients of the linear regression (black lines on Figure S1 - panels A and B). Variants from the entire range were considered for the MAF analysis and only variants above CADD score 20 were used, as this segment of the plot displayed a roughly linear relationship.

The same analysis was then performed on 100,000 gene panels, each containing the same number (62) of genes which were selected at random from the set of all genes captured by the enrichment kits (15,302). The distribution of slope coefficients obtained for these random panels are shown on Fig.S1 - panels C and D. The significance of the trends observed for the inflammasome gene panel was calculated as the proportion of randomly generated gene panels resulting in more extreme slope coefficients (single tailed). P-values for MAF by cohorts: MSFAM: 0.0002; MSS: 0.0011; combined MS cohort: 0.0005. P-values for CADD by cohorts: MSFAM: 0.0092; MSS: 0.0353; combined MS cohort: 0.0179.

The results show, that the trends of increased relative variant burden (in MS) with increased variant rarity and predicted pathogenicity are very unlikely to be observed when random genes were considered. Furthermore, amongst the 100,000 random gene panels, none achieved the trends observed for the inflammasome gene panel jointly for both MAF and CADD score. Additionally, a nearly equal representation of the opposing trends (density peaks around zero slope) for random gene panels demonstrate that the MS and control cohorts do not systematically differ in high-CADD-score or ultra-rare variant content, confirming that our results for the inflammasome gene panel were not due to the bias intrinsic to the dataset.

Supplementary Figure S1


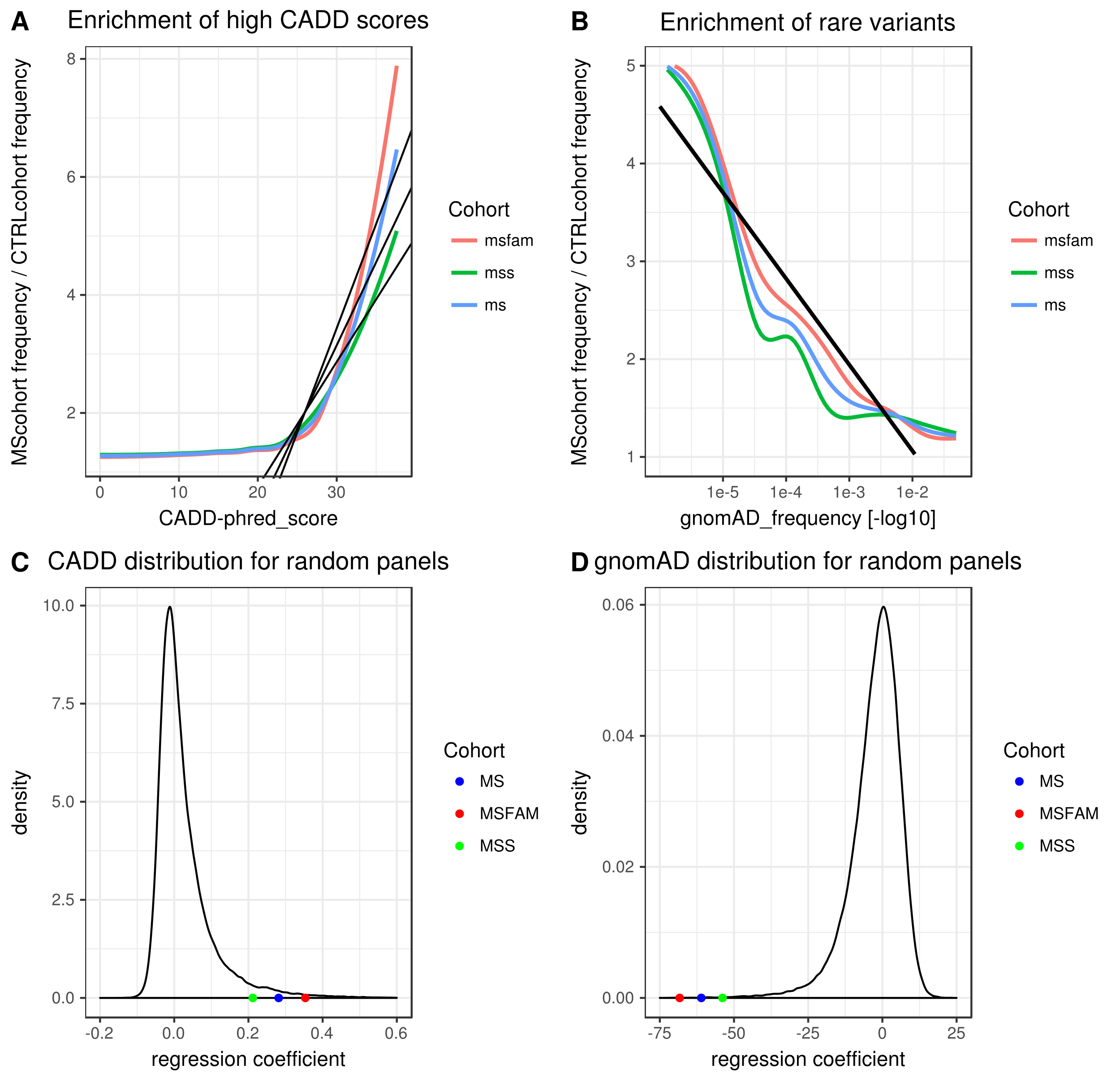


Supplementary Figure S1: Panels A and B: Relative variant burden with increased predicted pathogenicity and variant rarity. Panels C and D: The distribution of Slope coefficients obtained for 100,000 randomly generated panels and the slope coefficients obtained for the inflammasome gene panel (colored dots). Msfam: familial MS cohort, Mss: sporadic MS cohort, Ms: combined MS cohort.
